# Intrinsically Disordered Proteins in Bcl-2 Regulated Apoptosis

**DOI:** 10.3390/ijms11041808

**Published:** 2010-04-16

**Authors:** Gilles J. P. Rautureau, Catherine L. Day, Mark G. Hinds

**Affiliations:** 1 Walter and Eliza Hall Institute of Medical Research, 1G Royal Parade, Parkville 3052, Australia; 2 Department of Biochemistry, University of Otago, Dunedin 9054, New Zealand

**Keywords:** apoptosis, Bcl-2, BH3-only, intrinsically disordered protein, protein structure

## Abstract

Intrinsic cell death is mediated by interaction between pro-apoptotic and pro-survival proteins of the B-cell lymphoma-2 (Bcl-2) family. Members of this family are either intrinsically disordered or contain intrinsically disordered regions/domains that are critical to their function. Alternate splicing and post-translational modifications can determine the extent of these disordered regions and are critical for regulating Bcl-2 proteins. Conformational plasticity and structural transitions characterize the interactions within the Bcl-2 family, with conserved sequence motifs on both binding partners required for their molecular recognition.

## 1. Introduction

In response to intra-cellular stress signals cells initiate a programmed cell suicide known as apoptosis. Members of the Bcl-2 family of proteins are critical to the regulation of ‘intrinsic’ or mitochondrial initiated cell death [1]. The Bcl-2 family consists of members that either promote apoptosis, the pro-apoptotic proteins, or those that inhibit this action, the pro-survival proteins. Protein-protein interactions between these opposing factions mediate the life/death switch and it is thought the balance between the pro-survival and pro-apoptotic proteins decides cell fate [2]. As might be expected, their role as caretakers of cell fate ensures that the Bcl-2 family is highly regulated by a plethora of transcriptional, translational and post-translational mechanisms.

**Figure 1.**
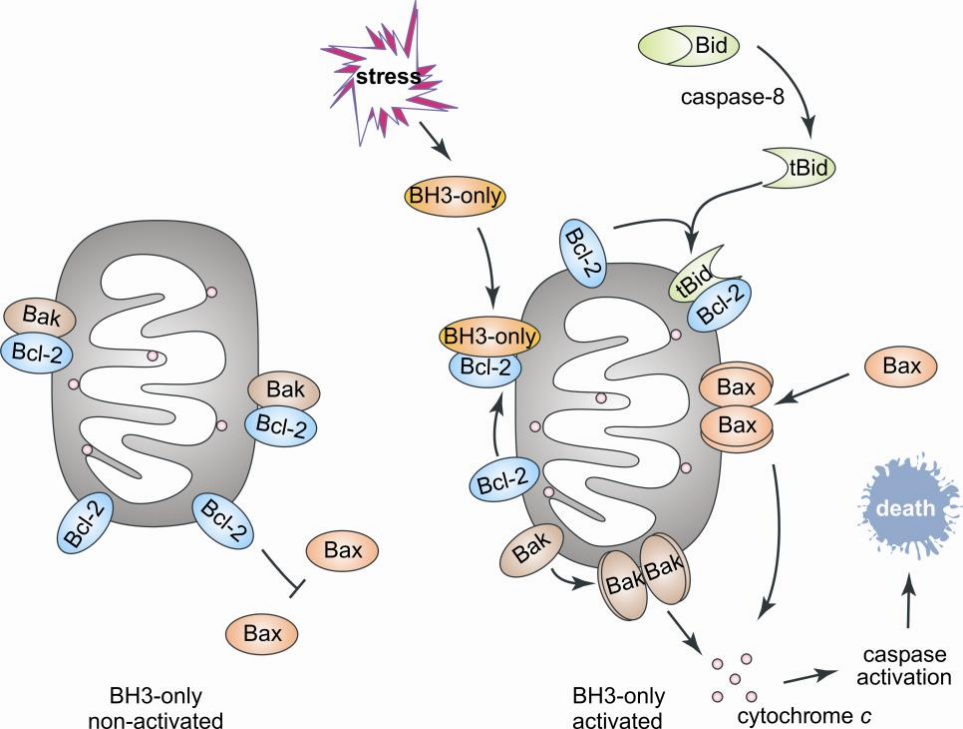
**General scheme showing the role of the Bcl-2 family in ‘intrinsic’ or mitochondrial activated apoptosis**. The BH3-only protein Bid links cell death receptor (‘extrinsic’) signaled apoptosis to intrinsic apoptosis. Bid is activated by proteolytic cleavage by caspase-8 in its IDR.

**Figure 2.**
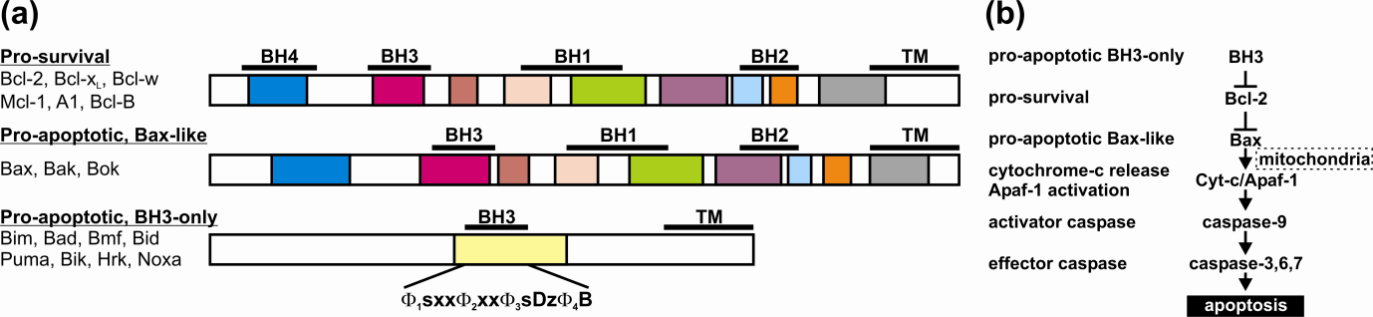
**The Bcl-2 family of proteins and their mode of action.** (**a**) The Bcl-2 family is split by structure and sequence into two groups: the multi-motif proteins that includes both pro-survival (Bcl-2, Bcl-x_L_, Bcl-w, Mcl-1, A1, Bcl-B) and pro-apoptotic members (Bax, Bak, Bok) that have an all helical Bcl-2 fold with IDRs and the IDP BH3-only proteins that only have a single BH3 motif (Bim, Bad, Bmf, Bid, Puma, Bik, Noxa, Hrk). The colored bars indicate the extent of helices and the location of BH motifs is indicated by black bars (**b**) Scheme showing the mode of Bcl-2 family action. The BH3-only proteins inhibit the pro-survival action allowing the Bax-like proteins to permeablize the mitochondrial outer membrane releasing cytochrome *c* to activate the caspase cascade that destroys the cell.

The sequence identity shared between members of the Bcl-2 family ranges between 3 and 40% but is typically <20%. Bcl-2, Bcl-x_L_ and Bcl-w are the most similar with Bcl-2/Bcl-x_L_ having 39.9% sequence identity. Among the BH3-only proteins Bim and Bmf have 20.4% shared identity, while the multi-motif pro-apoptotic protein Bax and BH3-only protein Hrk share only 3.2% identity. Although the sequence identity shared in the Bcl-2 family is low they all bear conserved sequence regions known as Bcl-2 homology (BH) domains or motifs and harbor up to four of these regions, BH1-BH4. The BH motifs are functionally important and underpin the interaction between Bcl-2 proteins [3]. The pro-apoptotic proteins either bear a single BH domain, the BH3-motif, and are known as the BH3-only proteins (members of this group include Bim, Bad, Bmf, Bid, Bik, Puma, Noxa and Hrk) or are structurally homologous to the pro-survival proteins Bcl-2, Bcl-x_L_, Bcl-w, Bcl-B, Bfl-1, and Mcl-1 and and are known as the multi-motif Bax-like proteins (Bax, Bak and Bok) [3] (Figures 1 and 2). In addition to bearing BH domains, Bcl-2 family proteins frequently have hydrophobic residues at their C-termini, identified as the ‘trans-membrane’ (TM) motif, which are important for localization to intra-cellular membranes such as the mitochondrial outer membrane (MOM), endoplasmic reticulum and the nuclear envelope [4].

In mammals, the Bcl-2 proteins control the integrity of the MOM by a still poorly characterized and controversial mechanism [5]. The BH3-only proteins act as the cellular sentinels that are activated in response to an apoptotic stimulus and they initiate the apoptotic process by binding in a hydrophobic groove located on the surface of the pro-survival proteins. This interaction liberates the pro-apoptotic Bax-like proteins, which effect apoptosis by releasing factors such as cytochrome *c* from the mitochondrial inter-membrane space into the cytosol (Figure 1). Gene knockout studies have shown that intrinsic apoptosis is dependent on the presence of Bax-like proteins and deletion of *bax* and *bak* render cells resistant to apoptotic stimuli [6]. Once released, cytochrome *c* interacts with cytosolic scaffold proteins promoting dimerization and activation of caspases, cysteine aspartyl proteases. Activated caspases execute a proteolytic cascade and destroy the cell from within, leading to its death, breakdown and phagocytosis [1].

Aberrant regulation of apoptosis has been directly linked to many diseases and is one of the hallmarks of cancer [7]. The Bcl-2 family proteins key role in determining cell fate has led to an intensive effort to understand their mode of action with the aim of therapeutic intervention [8]. These studies have uncovered important roles for unstructured motifs with the action of the Bcl-2 family as well as many of their up- and down-stream effectors, depending upon residues that do not have well-defined conformation. In addition covalent modification of BH3-peptides is being used to conformationally restrict these peptides to improve their *in vivo* proteolytic stability and the potency against tumors to explore their utility in cancer treatments [9]. Here we review the roles of intrinsically disordered proteins (IDPs) and intrinsically disordered regions (IDRs) and their interactions in the Bcl-2 family.

## 2. Bcl-2 Proteins Are IDPs, or Contain IDRs

**Figure 3.**
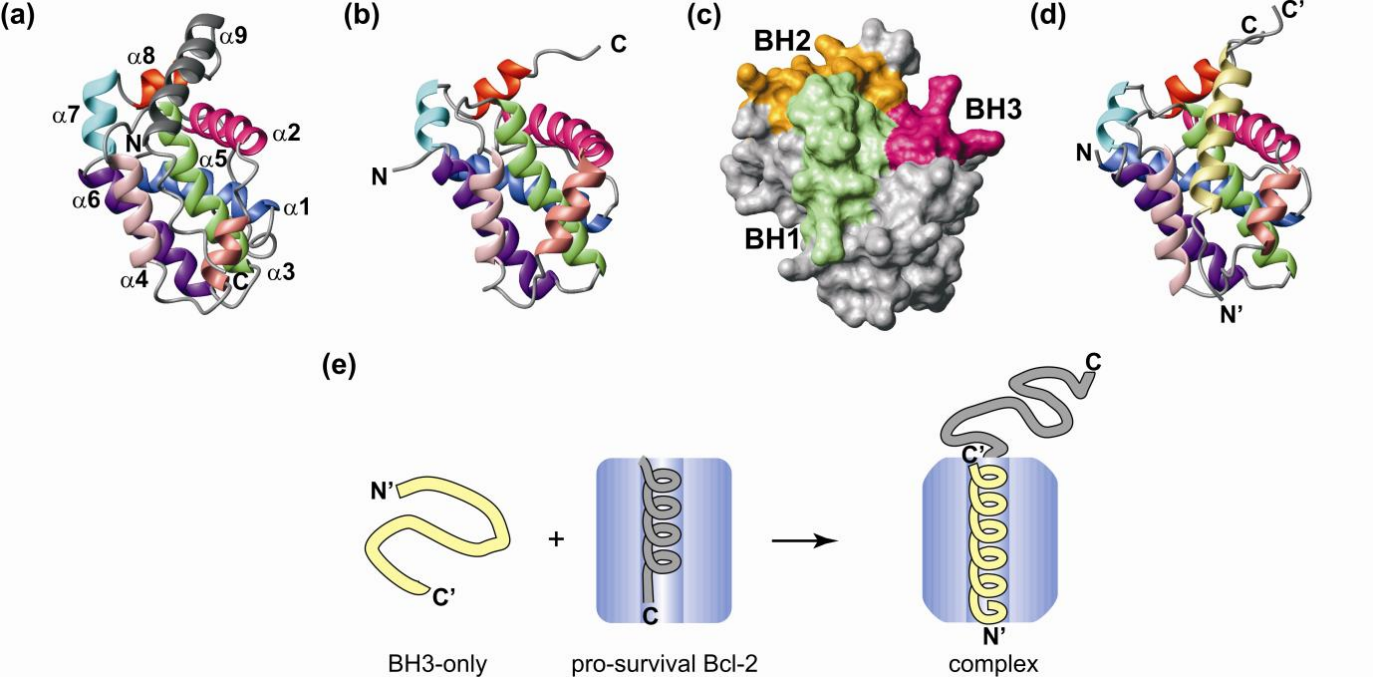
**Structures of pro-survival proteins and their BH3-only complexes.** (**a**) Ribbon diagram of Bcl-w (PDB: 1O0L) [16]. The helices are labelled α1-α9. (**b**). Ribbon diagram of unliganded C-terminally truncated Mcl-1 (1WSX) showing the binding groove [17]. (**c**) The BH1, BH2 and BH3 motifs are brought into close proximity on Mcl-1 by the Bcl-2 fold of the pro-survival proteins to provide a surface exposed hydrophobic groove that binds the BH3 motif of a BH3-only protein as an α-helix. (**d**) The IDP Noxa bound in the groove of Mcl-1 (2ROD) [27]. (**e**) Schematic of BH3-only protein binding a pro-survival protein. The BH3-only proteins are IDPs that displace a structured C-terminal helix to bind and fold in a hydrophobic groove of the pro-survival protein. This displacement generates an IDR at the C-terminus of the pro-survival protein.

The first structures in the Bcl-2 family became available over a decade ago [10–12] and a number of Bcl-2 structures and their complexes have since been solved. These have recently been incorporated into a sequence-structure database, BCL2DB [13]. The solution structure of C-terminally truncated Bcl-x_L_ [10] revealed a helical bundle structure with a long inter-helical loop of ∼60 residues that connects helices α1 and α2. The Bcl-x_L_ structure, as well as structures of other Bcl-2 proteins, demonstrated that both the pro-survival and Bax-like proteins share this helical fold, now described as the Bcl-2 fold. The Bcl-2 fold consists of a helical bundle where 7 amphipathic helices (α1-α4, α6-α8) pack against a central solvent inaccessible hydrophobic helix (α5) [3] (Figure 3). In addition Bax [14] and Bcl-w [15,16], that were obtained as soluble proteins without requiring such extensive truncation of their C-terminal residues, bear a ninth helix (α9), containing the TM region, that lies in a hydrophobic groove created by helices α2-α5 and α8. Biochemical data suggested that the TM region blocks access to the binding site in other pro-survival proteins [16]. Disordered residues are found in the long α1-α2 inter-helical loop of Bcl-2 and Bcl-x_L_, other multi-motif Bcl-2 proteins have a short mobile loop of approximately 12 residues in the corresponding position. Uniquely, Mcl-1 has an extended N-terminal region predicted to be unstructured and this region of ∼160 residues [17] contains regulatory elements such as two PEST sequences [18], (sequences enriched in proline [P], glutamic acid [E], serine [S] and threonine [T] [19]), which are in part responsible for regulating its degradation [20].

In contrast to their pro-survival and Bax-like relatives the BH3-only proteins lack long-range structure [21]. Sequence analysis, circular dichroism (CD), nuclear magnetic resonance (NMR) spectroscopic studies and biochemical evidence such as proteolytic susceptibility demonstrate that the BH3-only proteins are IDPs and even in the presence of their binding partners only the short BH3-motif has a defined structure [21,22]. Bid differs from other BH3-only proteins and has a Bcl-2 like fold structure with an IDR of 43 residues connecting helices α1 and α2 [23,24]. However, Bid is activated by proteolytic cleavage at a conserved site within the α1-α2 loop to generate a p7 and p15 fragment of 7 and 15 kDa respectively. The C-terminal p15 fragment of Bid is known as truncated Bid, or tBid and contains the BH3 motif. tBid has partial α-helical secondary structure, but it is in dynamic conformational exchange consistent with a molten globule state [25]. tBid is thus an example of an IDP that can be generated by post-translational modification of a folded protein.

## 3. Structural Transitions Characterize Bcl-2 Interactions

One of the central features underlying interactions in the Bcl-2 family is their conformational mobility [3]. The BH3-motif of IDPs like Bim, Bad, Bmf and tBid folds into an α-helix on binding in a hydrophobic groove formed from residues in helices α2-α5 and α8 on the pro-survival protein [11] (Figure 3). The exact intermolecular contacts and changes in geometry of the partners upon interaction are dependent on the specific components of the complex [17,26]. In the uncomplexed state, the groove of Bcl-x_L_ is in a closed state with α3 and α4 well-formed helices that are parallel and closely packed [22]. On binding Bim, helix α4 of Bcl-x_L_ rotates to accommodate the ligand and the α3 residues no longer form a regular α-helix [22]. In contrast, Mcl-1 has a binding site that is similar in the presence and absence of the ligand [27,28].

Although the exact interactions between BH3-only proteins and their pro-survival binding partners vary between complexes, a number of common features are apparent due to the presence of the 13 residue motif, Φ_1_sxxΦ_2_xxΦ_3_sDzΦ_4_B in all ligands. The conserved leucine at position Φ_2_ and aspartic acid residue at position D defines the BH3 motif [27]. The four hydrophobic residues, Φ_1_-Φ_4_, form a hydrophobic surface on the amphipathic BH3-helix. The other positions are: x is any residue, s small residues (G, A or S), z is normally an acidic residue and B a hydrogen bond acceptor. This short peptide motif binds and folds as an amphipathic helix in the hydrophobic groove of the pro-survival protein burying the hydrophobic residues Φ_1_–Φ_4_ in the interface. The conserved aspartic acid, D, forms a salt bridge with a conserved arginine in the BH1 motif of the pro-survival protein. Analysis of Bim and tBid binding to Bcl-w [21] and Bcl-x_L_ [25] show that helical structure is only induced over the BH3 motif and outside this region the sequence remains poorly ordered.

Using a conformationally restricted Bim peptide, that maintained a higher helical propensity and affinity than the unmodified BH3-peptide, it was discovered that Bim binds Bax through its hydrophobic surface [29]. Curiously, although Bax is structurally homologous to the pro-survival proteins [14] the Bim binding site was not mapped to the equivalent binding groove, but rather to the opposite face. The Bim binding surface on Bax was provided by helices α1 and α6 and there was little change in the Bax conformation on binding Bim. The surface provided by α1 and α6 on Bax does not rely on interaction with highly conserved residues like the groove and demonstrates the potential of multi-motif proteins to provide alternate binding surfaces and the BH3-only protein to adapt to these surfaces. Bax is normally cytosolic but localizes to mitochondria on initiation of apoptosis [30]. Bim binding Bax may provide the trigger that activates this translocation [29].

The C-terminal TM helix of Bcl-w, α9, like that of full-length Bax [14] lies in the hydrophobic groove used by the pro-survival proteins to bind their pro-apoptotic counterparts [15,16]. Binding a BH3-only protein displaces the C-terminal residues from the groove of Bcl-w and they become unstructured in solution [15,16] forming a new IDR (Figure 3). The ligand induced order-disorder transition of the TM residues appears to be driven by a disorder-order transition in the BH3 motif of the ligand and may be the initial step that leads to membrane integration of the pro-survival protein. The TM residues are necessary for correct localization to the membrane on BH3-only protein binding [31] and studies suggest correct localization is critical as Bcl-w is not fully protective in their absence, even though binding to BH3-only proteins is retained [16].

## 4. Bcl-2 Protein Structural Plasticity and Multiple Binding Partners

BH3-only proteins have a striking ability to interact with the binding grooves of multiple pro-survival proteins [32–34]. Like other protein-protein interactions those between an IDP and structured protein are entropy-enthalpy compensated [27] indicating that solvation plays an important role in binding [35]. While some BH3-only proteins bind all pro-survival proteins with approximately equal (high) affinity, others bind only selected pro-survival proteins and their capacity to promote apoptosis is limited [34]. The binding affinities can be remarkably tight, with dissociation constants typically in the nanomolar range [26,27,34]. Coupled folding and binding of IDPs provides a mechanism that fine tunes binding specificity to multiple targets. Conformational mobility permits local conformational adaptation for binding [36,37] and the ability of BH3-only proteins to engage multiple binding partners is in part due to their significant structural plasticity [17,26]. Bid, Puma and Bak BH3-peptides bind A1 with dissociation constants, K_d_ < 1–3 nM, while Bmf has an approximately 100 fold lower affinity with a K_d_ of 180 nM [26]. The majority of the intermolecular contacts for these four A1 complexes are identical. The highly conserved BH3 leucine and invariant aspartate of the BH3 motif make key contacts. The leucine is buried in a hydrophobic pocket provided by a cluster of highly conserved hydrophobic residues, while the aspartate makes a salt bridge with the conserved arginine in the BH1 motif. In the case of the low affinity A1:Bmf interaction the Bmf-BH3-motif packs in the binding groove tightly, but strikingly, the inter-molecular salt-bridge with the conserved aspartate is absent, instead it forms an intra-molecular salt bridge with a nearby arginine [26]. The importance of the salt bridge for high affinity interactions was also demonstrated for interaction of Bak BH3-peptide with Bcl-x_L_, where mutation of the aspartate in Bak BH3 lead to significantly weaker binding [11]. Therefore, although hydrophobic contacts are important, charged and hydrogen bonding also play an important role in BH3 interactions.

**Figure 4.**
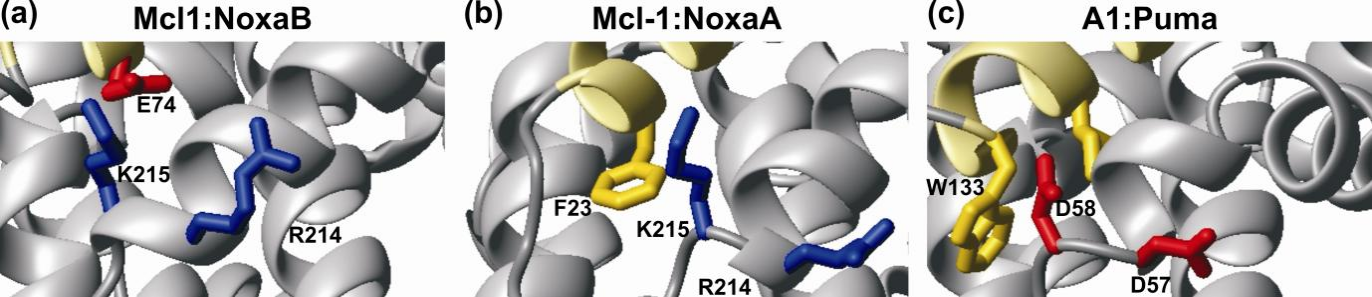
**Close-up view of differences between the A1 and Mcl-1 grooves. (a)** The presence of a basic patch provided by residues R214 and K215 in the α3-α4 loop in Mcl-1 accommodates the presence of an acidic residue (E74) in the BH3 domain of mouse NoxaB (PDB: 2NLA) [27]. **(b)** The same view of the Mcl-1:NoxaA complex (PDB: 2ROC) where a hydrophobic residue (F23) occupies a similar position to E74 in (a). **(c)** An acidic patch formed by residues D57 and D58 occupies the structurally equivalent position in A1 (PDB: 2VOF) to the basic patch in Mcl-1. NoxaB does not bind A1 [26]. The ribbon of the BH3 ligand is colored yellow. Basic residues are shown in blue and acidic residues in red.

Several studies have indicated the importance of the conformational mobility of the pro-survival protein binding groove and pro-survival proteins are tolerant of sequence changes in the BH3-ligand. For example, Mcl-1 tolerates a wide range of residues at the Φ_1_ position of the BH3-motif while Bcl-x_L_ does not [27,38]. Novel peptide sequences selective for Mcl-1 were identified using phage display that showed Mcl-1 could tolerate some degree of change from the canonical BH3-sequence [39]. The interaction of Mcl-1 with Noxa illustrates that the sequence composition of the groove also contributes to selective BH3 binding. Mouse Noxa has two BH3-motifs differing at the N-terminal Φ_1_ residue, the C-terminal BH3 (NoxaB) has a glutamic acid residue instead of the usual hydrophobic residue. NoxaB, binds Mcl-1 but not A1, while the N-terminal Noxa-BH3 motif (NoxaA) has the usual hydrophobic residue at Φ_1_ and binds both A1 and Mcl-1 [27]. The Mcl-1 has a conserved basic patch in the α3-α4 loop that can compliment the negatively charged acidic residue E74, while in A1 at the structurally equivalent position has acidic residues (Figure 4). On binding ligand similar structural changes are induced in Mcl-1 that are almost independent of the particular BH3 peptide ligand bound [27] and mutant Bim BH3 ligands bound tightly to Mcl-1 with only minor structural changes to Mcl-1 [40]. Point mutations to groove residues on the BH3-binding binding surface of Mcl-1 had little effect on Bim binding [17]. Each of the pro-survival proteins appears to have subtly different binding grooves and the combination of the dynamics of the unstructured ligand and those of the receptor pro-survival protein determine their selectivity and affinity. The ability of IDPs and their receptors to form alternative inter-molecular contacts is an important characteristic of their interactions.

## 5. Alternate Splicing Leads to Structural Diversity

Alternate splicing is an important regulation mechanism in the Bcl-2 family and not only regulates Bcl-2 protein levels but also their interactions. New molecular species are generated by alternate splicing that have modified structures and biological behavior. The presence of multiple splice variants for most Bcl-2 family members [41] increases the complexity of apoptosis signaling. The pro-apoptotic signal of the BH3-only proteins is contained within the short ∼16 residue BH3-motif [42] and exists on a single exon [43]. Most, if not all, BH3-only proteins like Bim [44–46], Bid [47], Bad [48,49], Bmf [50,51], Puma [52] and Noxa [53] have multiple splice variants. Regions flanking the BH3 motif in BH3-only proteins do not appear necessary for either pro-survival protein binding or specificity as the BH3 segment has essentially the full binding affinity for the pro-survival target [31]. Notably, peptides that span the BH3 motif bind with affinities and specificities [34] that predict the biology [54].

**Figure 5.**
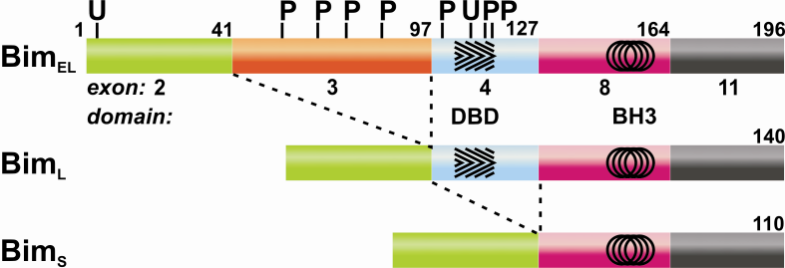
**Splice variants and post-translational modification sites in Bim.** The BH3 motif in the BH3-only proteins occurs on a single exon while the same region is shared with other motifs in the multi-motif proteins. The three main splice variants, short, long and extra long of Bim differ in exons 3 and 4. The corresponding sequence position is given above the bar and the exons are indicated. Marked on exon 4 is the DLC binding domain (DBD, hatched) and on exon 8 circles indicate the extent of the binding region of the BH3 motif. The potential phosphorylation sites are indicated with P and ubiquitylation sites with U.

Three major splice variants of Bim have been observed; Bim_S_, Bim_L_ and Bim_EL_, the short, long and extra long isoforms respectively and all are capable of initiating apoptosis [44,45] (Figure 5). Sequence analysis indicates all are IDPs [21] and all three isoforms bear the BH3-motif that is encoded by exon 8, however they have different activities depending on the other exons translated. Bim_L_ and Bim_EL_, for instance, have the binding site for the dynein motor complex protein dynein light chain 1 (DLC1) and are regulated by interaction with this protein [55]. Bmf also associates with a motor complex through binding a dynein light chain [56], but associates with the myosin V motor complex through binding DLC2 [57]. This interaction occurs through a short motif, (K/R)XTQT (X is any residue) [58] located on exon 4 that forms a complementary anti-parallel β-strand extending the β-sheet of the DLC protein. Association of Bmf and Bim with their respective DLC proteins compartmentalizes them [57] and like Bim_S_ the exon that encompasses the DLC binding motif in Bmf_S_ is not translated [51]. In addition to the three main isoforms of Bim, there are numerous other mRNA transcripts some of which are missing key exons but it is unclear if they are all translated to proteins [59]. At least three splice variants of Bid are known, and all would be expected to be IDPs like tBid, [47], yet they differ in their expression patterns, localization, regulation and apoptotic initiation. Unlike their structured counterparts IDPs are not reliant on a tertiary structure for their functionality and could be considered molecular ‘cut and paste’ frameworks. The unstructured nature of BH3-only proteins means that the splice variants can retain selected functionality and their prevalence may regulate specific apoptotic pathways. The development of unstructured regions through splice variants allows structure-independent changes in function that can modulate these molecules and impact on their ability to initiate apoptosis.

The BH1-BH3 motifs, or equivalent regions, in the pro-survival Bcl-2 proteins constitute the BH3-ligand binding surface and are required for their pro-survival activity [3]. Alternate splice variants of the multi-motif Bcl-2 proteins Bcl-2, Bcl-x_L_, Mcl-1, Bfl-1, Bax and Bok have been reported [41]. The BH3 motif is located on a single exon for Bax, Bak and Mcl-1, potentially making them targets of exon shuffling [43] while, in the case of Bok, it is split between two exons. In other multi-motif proteins either the BH1 or BH4 motif is found on the same exon as the BH3 motif. Many splice variants of these proteins lack the BH1 and BH2 motifs, but retain the BH3 motif. These proteins are therefore likely to lack a tertiary structure and may be pro-apoptotic by virtue of their exposed BH3 motifs. For example, Mcl-1S, a ‘short’ splice variant of Mcl-1, retains the N-terminal unstructured and BH3 motifs, but lacks the BH1, BH2 and TM motifs [60]. In contrast, the ‘extra short’ variant, Mcl-1ES retains BH1, BH2, BH3, the N-terminal residues and hydrophobic C-terminal region, but lacks the unstructured PEST sequences [61]. Mcl-1S and Mcl-1ES have been reported to have pro-apoptotic activity [39,60]. Alternate splicing thus provides an attractive mechanism for exposure of the BH3 motif and generation of an IDP with an altered apoptotic function.

In the case of Bax, a splice variant, Baxβ, has an extended C-terminal sequence that not only couples Baxβ to proteasomal degradation pathways but also renders it a more potent killer [62]. Splicing sites are generally more prevalent within IDRs as a splicing event within an IDR will minimize its effect on structured regions but broadens the functional and regulatory diversity [63]. However, not all splice sites fall in IDRs and a recent analysis indicates that many are located in structured regions and this may be a means of producing isoforms with new functional roles [64].

## 6. Generating New IDRs and Reactivity through Post-translational Modification

IDP levels are tightly controlled in the cell and kept at low levels by a combination of low protein synthesis and rapid degradation [65]. The control of protein activity and stability by post-translational modification is an important regulatory mechanism in apoptosis [66]. Characterized modifications in the Bcl-2 family include phosphorylation, ubiquitylation, caspase cleavage and deamidation [67]. These modification sites are largely confined to the IDRs, indicating the importance of these regions in regulating protein function.

Bim and Bad illustrate the complexity of post-translational control of BH3-only proteins and indicates their ability to interact with multiple post-translational modifying proteins. In a healthy cell Bad is inactivated by phosphorylation at multiple sites (see [67] for a summary) and sequestered to cytoskeletal 14-3-3 proteins that inhibit its action [68]. Many kinases (such as PKA, Akt/PKB or RSK) can phosphorylate and inactivate Bad and conversely, phosphatases (such as calcineurin), can dephosphorylate and reactivate its pro-apoptotic activity [69,70]. Phosphorylation by JNK at specific serines activates Bad by inhibiting its interaction with 14-3-3 proteins [71,72], but phosphorylation at other serines lowers its pro-apoptotic activity although it retains the ability to interact with Bcl-x_L_ [73]. Like Bad, post-translational modification at multiple sites modulates Bim activity (Figure 5) and each isoform may be differentially modified [74,75]. The proline directed kinase ERK1/2 phosphorylates Bim_EL_ at multiple sites on exon 3 (Figure 5) targeting it for proteasomal degradation [76] while the absence of the same exon in Bim_S_ and Bim_L_ renders these isoforms less sensitive to ERK1/2 phosphorylation [74,75,77]. The kinase Akt also phosphorylates Bim_EL_ on exon 3, but at a site separate from that of ERK1/2, reflecting its different specificity [78]. Akt phosphorylated-Bim can bind 14-3-3 proteins and this ablates its pro-apoptotic activity [78]. Therefore depending on the cellular context, multiple post-translational events can regulate the activity of Bim, Bad and other BH3-only proteins requiring their interaction with multiple proteins.

Activation of Bid occurs by proteolytic cleavage at conserved sites in its α1-α2 IDR by proteases such as caspase-8 and Granzyme B, the latter used in the immune response by killer T-cells to activate intrinsic apoptosis to eliminate infected cells [79]. The proteolytic cleavage generates an IDP capable of neutralizing the activity of pro-survival proteins. In addition to exposing its BH3 motif for interaction with pro-survival Bcl-2 partners [25], caspase cleavage of Bid generates a potential myristoylation site at the newly generated N-terminal glycine residue of tBid that may trigger its mitochondrial translocation [80] and ultimately the permeabilization of the MOM [81]. In contrast, phosphorylation of other sites in the IDR of Bid by both casein kinases I and II has been shown to prevent cleavage by caspase-8 and protect against death receptor induced apoptosis [82].

Although characterized by their Bcl-2 helical bundle, the multi-motif Bcl-2 proteins also have IDRs. These unstructured regions present sites for conserved post-translational modifications that may either inhibit or promote apoptosis [67]. Regulatory functions have been ascribed to the α1-α2 IDR of Bcl-x_L_ and Bcl-2 [83] and phosphorylation [84,85], deamidation [86] and proteolyic cleavage [87,88] in this region have all been associated with down regulation of pro-survival activity. Similarly, modification of residues in the IDR of Mcl-1 by proteolytic cleavage, phosphorylation or ubquitylation transforms its apoptotic activity [89]. Apart from the first 20 residues of Mcl-1 its IDR is variable [20], but the PEST sequences in the IDR are conserved and enhance its degradation contributing to its short half-life (1–5 h) [20,90]. In comparison, Bcl-2 has a half-life of ∼20 h [91]. In response to apoptotic stimuli Mcl-1 is rapidly upregulated and post-translational mechanisms are in part responsible for temporal control of protein levels [92]. Ubiquitin proteasome-mediated degradation also regulates the activity of Bcl-2, Bcl-x_L_ and Mcl-1 and ubiquitin modified lysines in Mcl-1 have been mapped to the N-terminal IDR and α1-α2 disordered loop [67,93].

The examples given above demonstrate the complexity of Bcl-2 family regulation through post-translational events. All members of the Bcl-2 family bear conserved sites for post-translational modification in unstructured regions that regulate their activity. These post-translational modifications connect apoptosis to other signaling pathways such as those signaled through MAP kinase [94] and also have the capability to interact with multiple other modifying proteins. Post-translational modification generates new molecular species with different interaction profiles and modified biological activity. Most post-translational modifications are still poorly characterized with neither the modification sites, temporal control nor the physiological context in which specific mechanisms prevail is clear. However, the crucial role of these modifications in regulating intrinsic apoptosis is unambiguous.

## 7. Conclusions

Unstructured regions play important roles in every aspect of Bcl-2 regulated apoptosis. All Bcl-2 family members either contain IDRs, like Bcl-2, Bcl-x_L_ and Mcl-1, or are IDPs, such as Bim, Bad and Bmf. The unstructured regions of Bcl-2 proteins are targets for post-translational regulation through interaction with enzymes that regulate their levels and capacity to interact with other members of the Bcl-2 family. Although the significance of many aspects of the post-translational modifications have yet to be established, collectively the multiplicity of regulatory processes that act through the unstructured regions of the Bcl-2 family attests to their importance in controlling Bcl-2 activity. The unstructured regions allow recognition by other proteins that is highly specific, but of low affinity. A key interaction regulating apoptosis that has been well investigated is that between pro-survival proteins and their BH3-only counterparts which fold and bind tightly as a helix within the groove of a pro-survival protein. Although it is thought hydrophobic interactions are overriding for IDPs [95], polar interactions make significant contributions to BH3-only binding [27]. The unstructured nature of the BH3-only proteins and their tight regulation is consistent with their position as initiators in apoptotic pathways and many have the ability to act promiscuously. This promiscuity coupled with protein compartmentalization, splice variation, post-translational modifications and degradation increases the complexity in cell death signaling and is likely to reduce the chance of inadvertently initiating a fatal signaling event [65]. Together, the interplay between the unstructured and structured components of the Bcl-2 family is crucial for determining cell fate.
